# Supplementary material for: Escalating risk-taking is linked to emotional habituation
Source: Commun Psychol. 2025 Sep 29;3:139. doi: 10.1038/s44271-025-00319-1 (PMC12479343; doi:10.1038/s44271-025-00319-1)
Supplement: Supplementary file 2 — Supplementary Information [file 44271_2025_319_MOESM2_ESM.pdf]

Supplementary Information

Escalating risk-taking is linked to emotional habituation

Hadeel Haj-Ali, Moshe Glickman, & Tali Sharot

(a)

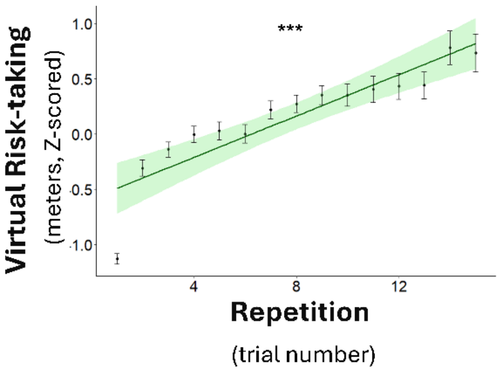

(b)

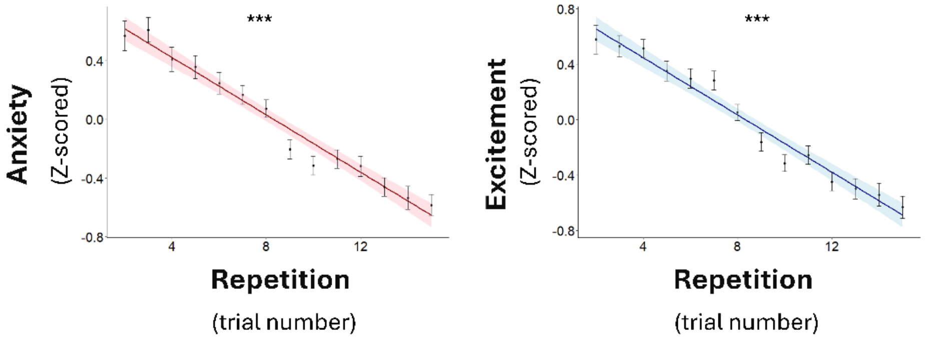

Supplementary Figure.1. Risk-taking escalates with repetition and corresponding emotions habituate (excluding trials where subjects stepped off the plank). This analysis excludes trials in which participants experienced a virtual fall. The results remain the same as reported in the main text. **(a)** Virtual risk-taking escalated such that participants walked further down the plank as trials progressed (linear mixed model predicting risk-taking from trial number as fixed effects with random slopes and intercepts: β = 0.209, t (50.92) = 11.53, p < 0.001). **(b)** Self-reported anxiety decreased as did excitement. In particular, we ran two linear mixed models predicting each emotional response (anxiety, excitement) on each trial from the trial number as a fixed factor with random slopes and intercepts. This analysis revealed that anxiety (β = -0.06, t (159.58) = -9.01, p < 0.001), and excitement (β = -0.07, t (159.49) = -9.73, p < 0.001), habituated with repetition. These findings were also true when controlling for distance traveled on each trial by adding it as another fixed variable with random slopes (anxiety: β = -0.06, t(152.02) = -8.51, p < 0.001; excitement: β = -0.07, t (152.96) = -10.12, p < 0.001). Dark colored regression lines represent the linear model prediction with the shaded color areas corresponding to the 95% confidence intervals. Scores are standardized within each subject for visualization purposes. Each dot represents the average Y scores across all participants on each trial. Error bars = standard error of the mean. ***p < .001.

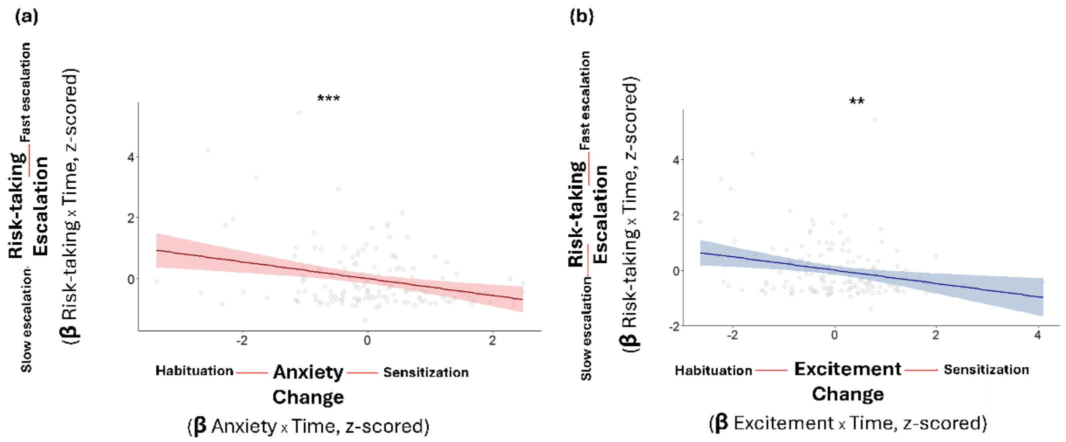

Supplementary Figure .2. Faster emotional habituation is associated with faster risk escalation (excluding trials where subjects stepped off the plank). This analysis excludes trials in which participants experienced a virtual fall. The results remain the same as reported in the main text. ***(a)*** Individuals with greater anxiety habituation (defined as a more *negative* relationship between anxiety and trial number) showed the fastest risk escalation (β = -0.27, t (140) = -3.44, p < 0.001). (**b**) Across subjects, those who exhibited a faster pace of excitement habituation (defined as a more ***negative*** relationship between excitement and trial number) showed faster risk escalation (β = -0.23, t (143) = -2.91, p = 0.004). Each dot represents a subject. Linear model prediction is represented by the bold line with the shaded areas corresponding to the 95% confidence bounds. **p < 0.01, **p < 0.01.

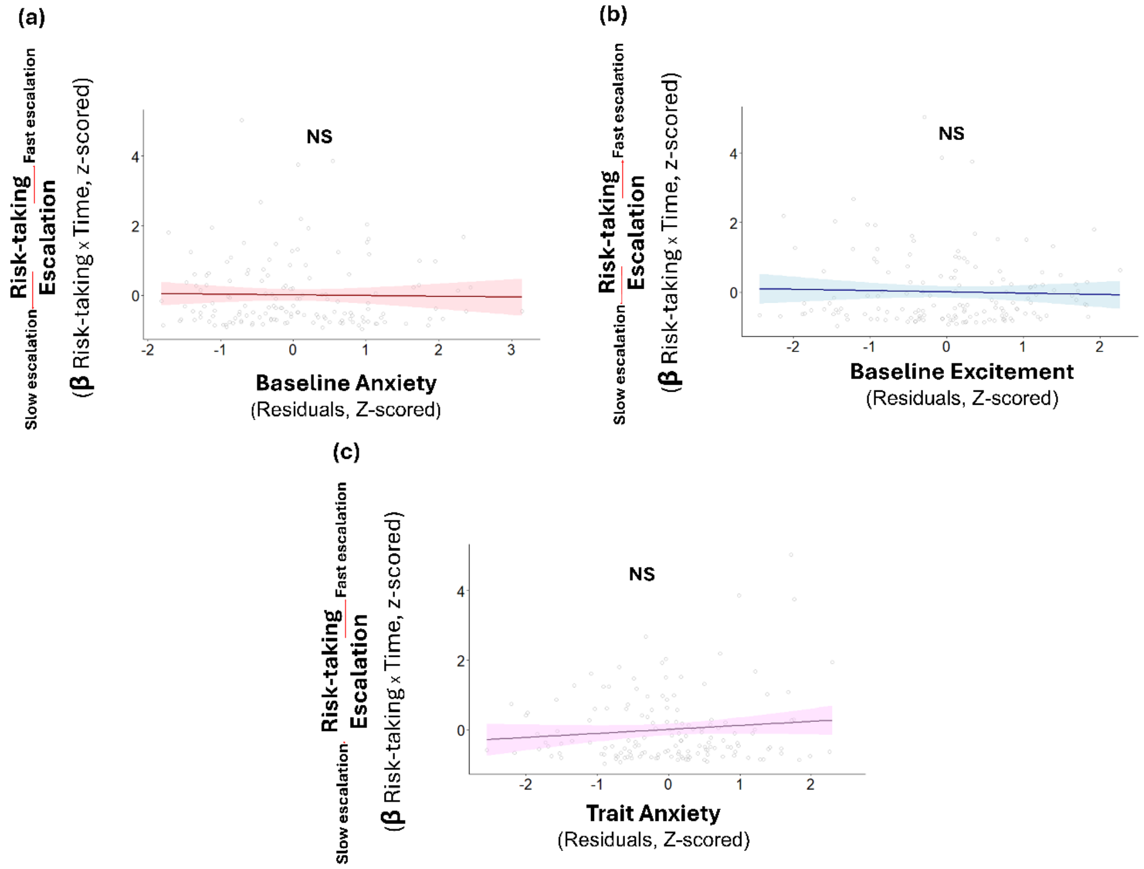

Supplementary Figure .3. Emotion Habituation, but not baseline emotions nor trait anxiety, predicts risk-taking escalation (excluding trials where subjects stepped off the plank). This analysis excludes

trials in which participants experienced a virtual fall. The results remain the same as reported in the main text. **(a)** Baseline anxiety reported prior to task exposure was not associated with the rate of risk escalation. Specifically, entering both baseline anxiety and anxiety habituation into a model predicting risk escalation revealed no significant effect of baseline anxiety ($\beta = 0.002$, $t(139) = 0.065$, $p = 0.948$, $BF_{10} = 0.03$), with the Bayes Factor providing strong evidence in favor of the null hypothesis. In contrast, anxiety habituation remained a significant predictor of risk escalation ($\beta = -0.27$, $t(139) = -3.16$, $p = 0.001$). **(b)** Baseline excitement reported prior to task exposure was also not associated with the rate of risk escalation. Specifically, entering both excitement habituation and baseline excitement into a model predicting risk escalation revealed a significant effect of excitement habituation ($\beta = -0.23$, $t(142) = -2.77$, $p = 0.006$), but not of baseline excitement ($\beta = 0.01$, $t(142) = 0.35$, $p = 0.726$, $BF_{10} = 0.03$), again supporting the null hypothesis. **(c)** Trait anxiety levels did not predict risk escalation, as revealed by a linear regression analysis ($\beta = 0.05$, $t(143) = 0.61$, $p = 0.538$, $BF_{10} = 0.212$), with the Bayes Factor providing evidence in favor of the null. Dark-colored lines represent the linear model prediction, with shaded areas corresponding to 95% confidence intervals. Each dot represents one subject. NS – not significant.
