# Supplementary material for: Escalating risk-taking is linked to emotional habituation
Source: Commun Psychol. 2025 Sep 29;3:139. doi: 10.1038/s44271-025-00319-1 (PMC12479343; doi:10.1038/s44271-025-00319-1)
Supplement: Supplementary file 3 — Reporting summary [file 44271_2025_319_MOESM3_ESM.pdf]

Corresponding author(s): Hadeel Haj-Ali and Tali Sharot

Last updated by author(s): Aug 28, 2025

Reporting Summary

Nature Portfolio wishes to improve the reproducibility of the work that we publish. This form provides structure for consistency and transparency in reporting. For further information on Nature Portfolio policies, see our Editorial Policies and the Editorial Policy Checklist.

Statistics

For all statistical analyses, confirm that the following items are present in the figure legend, table legend, main text, or Methods section.

n/a	Confirmed	
☐	☒	The exact sample size (*n*) for each experimental group/condition, given as a discrete number and unit of measurement
☐	☒	A statement on whether measurements were taken from distinct samples or whether the same sample was measured repeatedly
☐	☒	The statistical test(s) used AND whether they are one- or two-sided<br>*Only common tests should be described solely by name; describe more complex techniques in the Methods section.*
☐	☒	A description of all covariates tested
☐	☒	A description of any assumptions or corrections, such as tests of normality and adjustment for multiple comparisons
☐	☒	A full description of the statistical parameters including central tendency (e.g. means) or other basic estimates (e.g. regression coefficient) AND variation (e.g. standard deviation) or associated estimates of uncertainty (e.g. confidence intervals)
☐	☒	For null hypothesis testing, the test statistic (e.g. *F*, *t*, *r*) with confidence intervals, effect sizes, degrees of freedom and *P* value noted<br>*Give P values as exact values whenever suitable.*
☐	☒	For Bayesian analysis, information on the choice of priors and Markov chain Monte Carlo settings
☒	☐	For hierarchical and complex designs, identification of the appropriate level for tests and full reporting of outcomes
☐	☒	Estimates of effect sizes (e.g. Cohen's *d*, Pearson's *r*), indicating how they were calculated

Our web collection on statistics for biologists contains articles on many of the points above.

Software and code

Policy information about availability of computer code

Data collection	Data was collected using an already exisiting Virtual Reality Task. We used the virtual Richie's Plank Experience(https://www.meta.com/en-gb/experiences/richies-plank-experience/1642239225880682/?srsltid=AfmBOoqLGxFrT8veSId-KVRMTqNeFkYxZuXXP5Swmou5wHoIl_ACnmHi), developed by Toast and obtained from the Meta application store, and streamed on a Meta Quest 2 VR headset. All subjects provided written consent before the experiment and were paid £9/hr for their participation.
Data analysis	Data preprocessing and analyses were performed in R and RStudio (R Core Team, 2023; Posit Team, 2023). Bayesian analyses were run in JASP software (JASP team, 2023). Linear mixed effects were run with afex package (Singmann et al., 2016). Data visualization was created using the ggplot R package. We calculated the Bayes Factor (BF10) for each null effect, which measures the ratio of evidence in favor of the alternative hypothesis compared to the null hypothesis, given the data (Dienes, 2014).

For manuscripts utilizing custom algorithms or software that are central to the research but not yet described in published literature, software must be made available to editors and reviewers. We strongly encourage code deposition in a community repository (e.g. GitHub). See the Nature Portfolio guidelines for submitting code & software for further information.

Data

Policy information about availability of data

All manuscripts must include a data availability statement. This statement should provide the following information, where applicable:

- Accession codes, unique identifiers, or web links for publicly available datasets
- A description of any restrictions on data availability
- For clinical datasets or third party data, please ensure that the statement adheres to our policy

We used the virtual Richie's Plank Experience(www.meta.com/en-gb/experiences/richies-plank-experience/1642239225880682/?srsltid=AfmBOoqLGxFrT8veSId-KVRMTqNeFkYxZuXXP5Swmou5wHoIl_ACnmHi), developed by Toast and obtained from the Meta application store, and streamed on a Meta Quest 2 VR headset. Data and code used to analyze the data are available online via https://github.com/affective-brain-lab/Risk-Escalation.git

Research involving human participants, their data, or biological material

Policy information about studies with human participants or human data. See also policy information about sex, gender (identity/presentation), and sexual orientation and race, ethnicity and racism.

Reporting on sex and gender	We did not analyze sex differences, as they were not relevant to our primary research question, which focuses on the phenomenon of risk escalation and the factors that explain individual differences in it. Consequently, during data collection, we did not account for sex differences or ensure an equal distribution of men and women, which would have been necessary to examine such effects.<br>Main Experiment: N = 160 (mean age = 21.8, SD = 4.69; 117 women, 43 men). Validation Test: N = 35 (mean age = 21.942, SD = 3.695; 25 women, 10 men); Control Experiment: N = 30 (mean age = 21.366, SD = 2.689; 25 women, 5 men).
Reporting on race, ethnicity, or other socially relevant groupings	Demographics data of subjects including race were collected in all three experiments (main experiment, validation, and control) but were not analyzed as they were not relevant to our primary research question.<br><br>Main Experiment: N = 160 ; 104 Asian, 37 White, 10 Other, and 9 African American/Black. Validation Test: N = 35 ; 23 Asian, 9 White, and 3 Other); Control Experiment: N = 30 ; 21 Asian, 5 White, 3 Other, and 1 African American/Black).
Population characteristics	See above.
Recruitment	Participants were recruited from the UCL SONA recruitment system (uclpsychology.sona-systems.com). Participants with a very significant fear of heights were advised not to sign up for the study, to avoid psychological trauma
Ethics oversight	Experimental Psychology Ethics Committee Application EP_2023_009

Note that full information on the approval of the study protocol must also be provided in the manuscript.

Field-specific reporting

Please select the one below that is the best fit for your research. If you are not sure, read the appropriate sections before making your selection.

☐ Life sciences ☒ Behavioural & social sciences ☐ Ecological, evolutionary & environmental sciences

For a reference copy of the document with all sections, see nature.com/documents/nr-reporting-summary-flat.pdf

Behavioural & social sciences study design

All studies must disclose on these points even when the disclosure is negative.

Study description	This study comprised a series of behavioral virtual reality tasks designed to examine whether risk-taking behavior escalates with repeated exposure, whether emotional responses (both negative and positive) habituate over time, and whether these phenomena are linked. We employed a within-subject repeated measures design, collecting a behavioral measure of risk-taking, quantified as the distance walked on a plank (in meters) during each trial, and self-reported emotions at the beginning of each trial. Over 15 trials, participants repeatedly engaged in a risk-taking VR task involving a high, suspended plank.<br><br>A validation study was conducted to determine if walking further along the plank was associated with increased perceptions of risk. This experiment followed a design similar to the main task but also required participants to report their perceived risk at various points along the plank.<br><br>Finally, we ran a control study to assess whether the observed escalation in risk-taking and emotional habituation could simply be attributed to motor learning. In this experiment, Riche's plank paradigm was used as in the main experiment, except that the virtual plank was placed at ground level.
Research sample	The sample included undergraduates students from University College London residents in the London area. Main Experiment: N = 160 (mean age = 21.8, SD = 4.69; 117 women, 43 men). Validation Test: N = 35 (mean age = 21.942, SD=3.695; 25 women, 10 men);

	Control Experiment: N = 30 (mean age = 21.366, SD=2.689; 25 women, 5 men).
Sampling strategy	A convenience sampling method was used, with participants recruited through UCL SONA system, an online platform that allows individuals to voluntarily participate in experiments for £9/hr (total of £6) or 1 university credit. All subjects provided written consent before the experiment and were paid £6 for their participation.
Data collection	Behavioral data on risk-taking were collected through manual inspection of videos capturing participants' walk and movement on the plank. Risk-taking was defined as the distance walked on the plank, ranging from 0 to 5.525 meters. The physical plank in the room was precisely aligned with the virtual plank viewed by participants, and measurements were scored at regular intervals of 5 cm. Three experimenters, including fully qualified and trained PhD student and research assistants, encoded the video data to ensure high rating accuracy. Additionally, emotion self-reports were provided verbally by participants and subsequently entered into an Excel spreadsheet by an experimenter.
Timing	March 2023 - October 2024
Data exclusions	One participant was excluded from the analysis of the main experiment due to VR malfunction.
Non-participation	No participants had dropped out or declined participation.
Randomization	Studies were conducted within-subject.

Reporting for specific materials, systems and methods

We require information from authors about some types of materials, experimental systems and methods used in many studies. Here, indicate whether each material, system or method listed is relevant to your study. If you are not sure if a list item applies to your research, read the appropriate section before selecting a response.

Materials & experimental systems

n/a	Involved in the study
☒	☐ Antibodies
☒	☐ Eukaryotic cell lines
☒	☐ Palaeontology and archaeology
☒	☐ Animals and other organisms
☒	☐ Clinical data
☒	☐ Dual use research of concern
☒	☐ Plants

Methods

n/a	Involved in the study
☒	☐ ChIP-seq
☒	☐ Flow cytometry
☒	☐ MRI-based neuroimaging

Plants

Seed stocks	*Report on the source of all seed stocks or other plant material used. If applicable, state the seed stock centre and catalogue number. If plant specimens were collected from the field, describe the collection location, date and sampling procedures.*
Novel plant genotypes	*Describe the methods by which all novel plant genotypes were produced. This includes those generated by transgenic approaches, gene editing, chemical/radiation-based mutagenesis and hybridization. For transgenic lines, describe the transformation method, the number of independent lines analyzed and the generation upon which experiments were performed. For gene-edited lines, describe the editor used, the endogenous sequence targeted for editing, the targeting guide RNA sequence (if applicable) and how the editor was applied.*
Authentication	*Describe any authentication procedures for each seed stock used or novel genotype generated. Describe any experiments used to assess the effect of a mutation and, where applicable, how potential secondary effects (e.g. second site T-DNA insertions, mosiacism, off-target gene editing) were examined.*
